# Efficacy and safety of manufactured Chinese herbal formula for cervical radiculopathy: protocol for a systematic review with meta-analysis and trial sequential analysis

**DOI:** 10.3389/fneur.2025.1608095

**Published:** 2025-09-11

**Authors:** Xiaohang Bao, Baohua Huang, Canmei Li, Weixiong Gan, Zhifei Li, Wei Xu, Yisheng Zhang, Bin Tang

**Affiliations:** The First Affiliated Hospital of Guangxi University of Chinese Medicine, Nanning, Guangxi, China

**Keywords:** cervical radiculopathy, manufactured Chinese herbal formula, meta-analysis, protocol, efficacy, safety

## Abstract

**Background:**

Manufactured Chinese herbal formulas (MCHFs) are Chinese patent medicine preparations made from a variety of herbal ingredients. These formulas are processed into specific dosage forms in accordance with defined prescriptions and manufacturing procedures, and are intended for the prevention and treatment of various diseases. As a type of commercialized Chinese patent medicine, MCHFs are listed and marketed in China upon approval by the National Medical Products Administration (NMPA). MCHFs can be used to treat cervical radiculopathy (CR) and alleviate its symptoms, but the efficacy of MCHFs compared to conventional oral drugs for the treatment of CR has not been thoroughly explored. Therefore, this meta-analysis aimed to assess and compare the efficacy of MCHF with that of conventional oral drugs for the treatment of CR.

**Methods:**

The meta-analysis was conducted following the guidelines outlined in the Cochrane Handbook and the Preferred Reporting Items for Systematic Review and Meta-Analysis Protocols (PRISMA-P) checklist. A comprehensive search was conducted in PubMed, the Excerpta Medica database (EMBASE), the Cochrane Central Register of Controlled Trials (CENTRAL), and three Chinese electronic databases, including the National Knowledge Infrastructure (CNKI), Wanfang Digital Periodicals (WANFANG), and the Chinese Science and Technology Periodicals (VIP) database. We limited the type of literature included to RCT, and there was no language restriction. Two independent reviewers used the NoteExpress tool to screen studies, extract data, and assess the quality of the studies. The global Visual Analog Scale (VAS) pain score measured at the end of the treatment period was the primary outcome. Secondary outcomes included the Neck Disability Index (NDI), the Japanese Orthopedic Association (JOA) score, the 36-Item Short Form Health Survey (SF-36) score, and adverse events. If feasible, a meta-analysis was conducted in Review Manager 5.4; otherwise, a descriptive analysis was performed. The grading of recommendations assessment, development, and evaluation (GRADE) approach was used to assess the evidence level of the meta-analysis for primary outcome measure and all secondary outcome measures, and trial sequential analysis (TSA) was performed for the primary outcome measure.

**Discussion:**

This predefined protocol is intended to enhance transparency, avoid future duplication of efforts, and generate reliable evidence regarding the efficacy and safety of MCHF in the treatment of cervical radiculopathy.

## 1 Introduction

Cervical radiculopathy (CR) is a prevalent form of degenerative cervical spine disease, accounting for approximately 60–70% of all cases of cervical spondylosis, with a rising global incidence (1, 2). It primarily results from age-related degenerative changes in the cervical vertebrae, leading to spinal canal narrowing and nerve root compression. These pathophysiological changes manifest clinically as neck pain, numbness, and/or motor weakness in the upper extremities (3, 4). Current treatment strategies for CR include both conservative and surgical interventions, with conservative management generally recommended as the first-line approach due to its demonstrated efficacy in the majority of patients (5).

Oral pharmacological therapy represents one of the primary approaches within the conservative management of cervical radiculopathy. Conventional oral medications include non-steroidal anti-inflammatory drugs (NSAIDs), vitamin B12, muscle relaxants, anxiolytics, prostaglandin analogs, and glucocorticoids (6).

In recent years, manufactured Chinese herbal formulas (MCHFs) have been increasingly utilized in China and have gained growing international recognition. MCHFs refer to standardized formulations of Chinese patent medicines composed of herbal ingredients. These are processed into specific dosage forms based on traditional prescriptions and modern pharmaceutical manufacturing practices and are officially approved by the China National Medical Products Administration (NMPA) for the prevention and treatment of various diseases. MCHFs are widely applied in clinical practice in China, and their therapeutic effects have also been reported in international studies (7, 8).

In clinical practice, non-pharmacological treatments for CR, such as Chinese therapeutic massage and traction therapy, have been well-documented (9, 10). However, the efficacy of these interventions may vary depending on the practitioner’s level of experience, and their implementation can be limited by practical constraints. Similarly, the prescription of traditional Chinese medicine (TCM) decoctions requires extensive clinical expertise, and improper formulation may result in suboptimal efficacy or even potential toxicity. Furthermore, decoctions are often inconvenient for patients to prepare and consume. In contrast, MCHF offers standardized production processes, thereby minimizing dependence on practitioner skill and enhancing clinical applicability. MCHF has undergone comprehensive pharmacological, toxicological, and clinical efficacy evaluations, providing a solid foundation for its safety and therapeutic potential (11). Therefore, we conducted an evidence synthesis to systematically evaluate the clinical effectiveness and safety profile of MCHF in the treatment of CR.

Despite the widespread clinical use of MCHF, their comparative efficacy relative to conventional oral drugs for CR has not been comprehensively evaluated. Therefore, we performed a systematic review and meta-analysis to assess and compare the clinical efficacy of MCHF versus conventional oral drugs in the treatment of CR.

## 2 Methods

### 2.1 Protocol registration

This systematic review protocol was registered in the International Prospective Register of Systematic Reviews (PROSPERO; registration number: CRD42024605787). The protocol was developed following the Preferred Reporting Items for Systematic Review and Meta-Analysis Protocols (PRISMA-P) guidelines (12).

### 2.2 Study objective

The objective of this systematic review was to evaluate the clinical efficacy and safety of MCHF in the treatment of CR. In addition, subgroup analyses were conducted for all outcome measures based on different types of control interventions.

### 2.3 Inclusion and exclusion criteria

We included randomized controlled trials (RCTs) in this review without language restrictions. The eligibility of studies was assessed based on the PICO framework (Population, Intervention, Comparator, Outcome).

#### 2.3.1 Inclusion criteria were defined as follows

- Population (P): Patients with a confirmed diagnosis of CR, characterized by signs and symptoms of nerve root compression or irritation (13).
- Intervention (I): Studies in which MCHF was compared with placebo, conventional oral drug, or the combination of MCHF and conventional oral drug versus conventional oral drug alone, regardless of dose or treatment duration. MCHFs refer to Chinese patent medicines approved by the NMPA.
- Comparator (C): Control groups included placebo, conventional oral drug alone, or in combination. Conventional medications are as follows:

- Non-steroidal anti-inflammatory drugs (NSAIDs): e.g., Celecoxib Capsules, Aceclofenac Sodium capsules.
- Muscle relaxants: e.g., Tizanidine Hydrochloride tablets, Eperisone Hydrochloride tablets.
- Analgesics: e.g., Loxoprofen Sodium and Codeine Sustained-release tablets.
- Neurotrophic agents: e.g., Mecobalamin tablets, Neroxon tablets.

d. Outcomes (O): The primary outcome was the Visual Analog Scale (VAS) for global pain at the end of treatment. Secondary outcomes included the Japanese Orthopaedic Association (JOA) Score, Neck Disability Index (NDI), and the 36-Item Short Form Health Survey (SF-36) assessed at the end of treatment, as well as the incidence of adverse events during treatment.

#### 2.3.2 Exclusion criteria included the following

- Studies in which MCHF was used in both the intervention and control groups.
- Studies involving combination therapies with other traditional or complementary modalities, such as other herbal medicines, acupuncture, moxibustion, manual therapy, tai chi, or yoga.
- Reviews, animal studies, or conference abstracts.
- Studies with insufficient data or duplicate publication.

### 2.4 Database and search strategies

We systematically searched PubMed, Excerpta Medica Database (EMBASE), the Cochrane Central Register of Controlled Trials (CENTRAL), and three Chinese electronic databases, including the China National Knowledge Infrastructure (CNKI), Wanfang Data (WANFANG), and the Chinese Scientific Journal Database (VIP), to identify randomized controlled trials (RCTs) of MCHF for the treatment of CR. We also searched for ongoing trials in ClinicalTrials.gov (www.clinicaltrials.gov), the EU Clinical Trials Register and the Clinical Trials Information System (CTIS) (www.clinicaltrialsregister.eu), and the WHO International Clinical Trials Registry Platform (ICTRP, www.who.int/clinical-trials-registry-platform).

**Figure 1:**
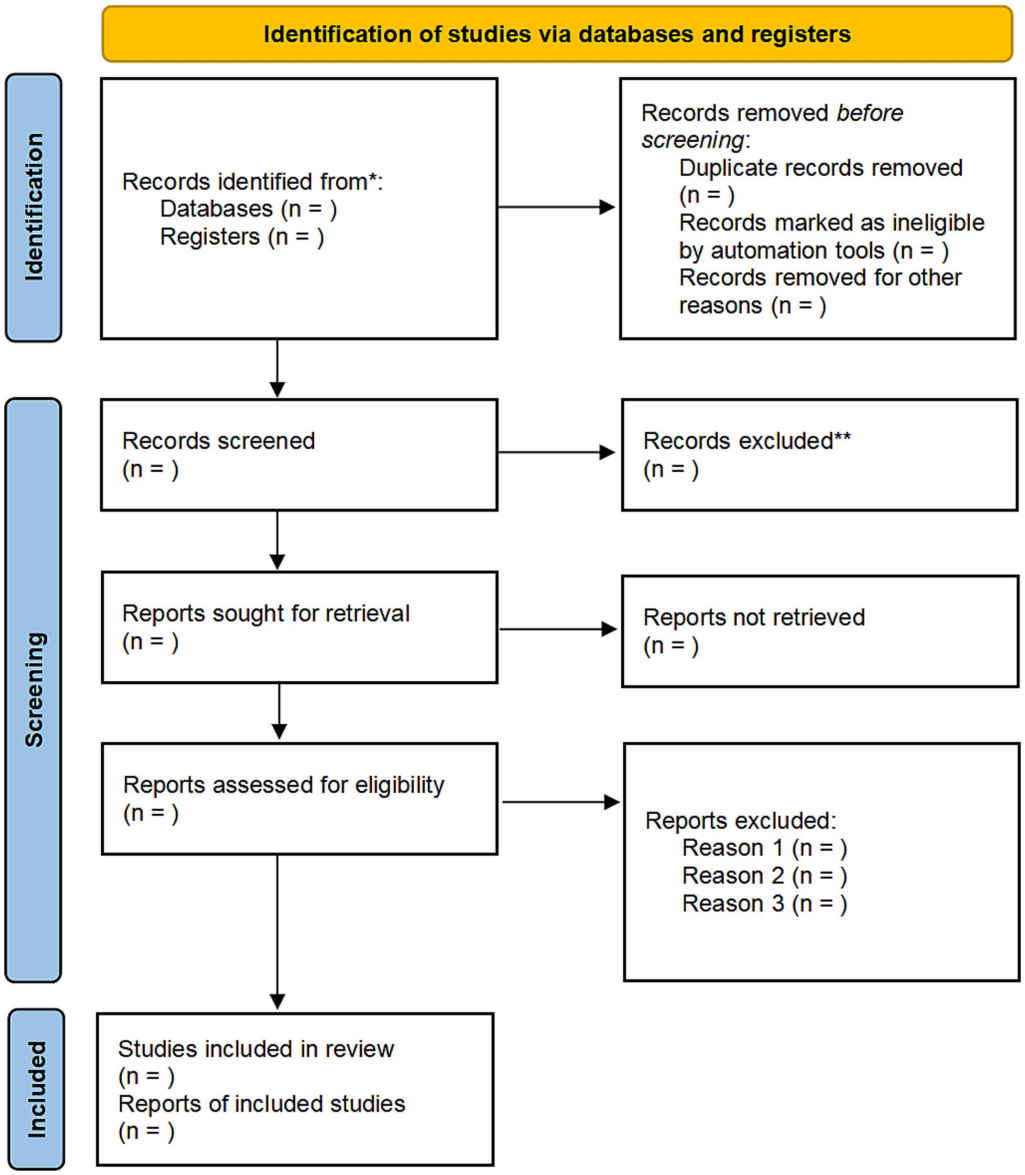
PRISMA flowchart for study selection.

The search strategy was piloted in October 2024, and all relevant studies were identified through electronic database searches from inception to September 30, 2024. A PRISMA flowchart outlining the study selection process is shown in Figure 1. Bin Tang and Xiaohang Bao prepared the content of the PRISMA flowchart; any discrepancies were reviewed and resolved by Zhifei Li. No restrictions were imposed on language or publication type. Unpublished studies were also sought. The searches were re-run prior to the final analysis. The detailed search strategy is provided in the Supplementary material.

### 2.5 Data extraction

After the literature search and removal of duplicates, two reviewers (Bin Tang and Xiaohang Bao) used NoteExpress software (version 4.0) for data extraction, and the extracted data were entered into an electronic database independently. The extracted data included the first author’s name, year of publication, sample size, population characteristics (age and sex of patients), interventions, duration of treatment, all study outcome assessments, and overall conclusions regarding the efficacy of MCHF. Details of the intervention and control groups included the name of the drug, dosage, therapeutic regimen, treatment duration, VAS, JOA, NDI, SF-36, and adverse events. Any discrepancies were resolved through discussion or, if necessary, by consulting a third reviewer (Baohua Huang). We extracted the outcome data after the last treatment, as presented by means and standard deviations. The outcome data were converted to means and standard deviations if described in other forms.

### 2.6 Risk of bias

The methodological quality of the included studies was independently assessed by Bin Tang and Xiaohang Bao using the Risk of Bias 2 (RoB 2) tool, following the criteria outlined in the *Cochrane Handbook for Systematic Reviews of Interventions* (14). The following domains were evaluated: bias arising from the randomization process, bias due to deviations from intended interventions, bias due to missing outcome data, bias in measurement of the outcome, and bias in selection of the reported result (15). We used the Shiny web application as a visualization tool for generating risk of bias (RoB2) figures specific to randomized controlled trials (16). Any discrepancies in the risk of bias assessments were resolved through discussion or consultation with a third reviewer (Wei Xu).

### 2.7 Data synthesis

We used Review Manager software (RevMan, version 5.4; Cochrane Collaboration) to conduct the data analysis. The minimum number of studies required for meta-analysis was two. Continuous outcomes were reported as mean differences (MD) or standardized mean differences (SMD) with 95% confidence intervals (CI). Binary outcomes were reported as risk ratios (RR) or odds ratios (OR), also with 95% CI. Heterogeneity among studies was assessed using the I^2^ statistic. If substantial heterogeneity was detected (I^2^ > 50%), a random-effects model was used to calculate the pooled estimates; otherwise, a fixed-effect model was applied. If quantitative synthesis were not appropriate, we would have described the type of summary planned.

### 2.8 Subgroup and sensitivity analyses

Subgroup analyses were conducted for each type of comparison, including MCHF versus placebo, MCHF versus conventional oral drug, and MCHF combined with conventional oral drug versus conventional oral drug alone. Further subgroup analyses were performed for the primary outcome according to the specific names of the MCHF within the same type of comparison. Additional subgroup analyses were explored to identify potential sources of heterogeneity, including year of publication, randomization methods, sample size, sex ratio, and duration of treatment. *p*-values for interaction (P_interaction_) were calculated to assess differences between subgroups.

Sensitivity analyses were focused on the primary outcome to assess the robustness of the results, using approaches such as changing the statistical model (fixed-effect vs. random-effects) and excluding specific types of studies. If the direction of the results remains unchanged, it indicates that the results are stable; if the direction changes, the results are deemed unstable.

### 2.9 Assessment of publication bias

If more than 10 trials are included, publication bias was assessed. The presence of small-study effects was evaluated both qualitatively and quantitatively. A qualitative assessment was performed by visual inspection of funnel plot symmetry, while a quantitative evaluation was conducted using Egger’s test and Begg’s test.

### 2.10 Qualitative analysis of evidence level

We used the Grading of Recommendations Assessment, Development, and Evaluation (GRADE) approach to assess the quality of evidence for both primary and secondary outcome measures in the meta-analysis. GRADE evaluated five domains: risk of bias, indirectness, imprecision, inconsistency, and publication bias (17, 18). The assessment was conducted using GRADEpro software (version 3.6.1; available at gradepro.org).

### 2.11 Trial sequential analysis

Trial sequential analysis (TSA) was performed for the primary effectiveness outcome. TSA was conducted using the Copenhagen Trial Unit’s dedicated software (version 0.9.5.10 beta, 2021 release) to evaluate the conclusiveness of the current evidence and inform the design of future studies. The analysis incorporated three key components: calculation of the heterogeneity-adjusted required information size (RIS), accounting for both between-study variance (using the DerSimonian–Laird estimator) and cumulative random error; construction of trial sequential monitoring boundaries using the O’Brien–Fleming *α*-spending function; evaluation of the cumulative evidence by comparing the accrued data with predefined thresholds (*α* = 5%, power = 80%). This methodology enabled a quantitative assessment of the reliability of meta-analytic conclusions. If the cumulative Z-curve crosses the trial sequential monitoring boundary, it indicates that firm evidence has been reached. If it crosses the futility boundary adjusted for the RIS, it suggests that further trials are unlikely to change the conclusion. However, if the Z-curve remains within the area of uncertainty, additional research is needed (19). In theory, robust evidence can be considered established when the Z-curve crosses either the monitoring boundary or reaches the required information size. Conversely, if neither is crossed, additional trials are likely needed to reach conclusive evidence.

## 3 Discussion

Due to the high risks associated with surgery, conservative treatment remains the preferred approach for CR, including physical therapy, oral medications, injections, and the use of cervical collars or braces (20, 21). Currently, there is no universally accepted first-line oral medication for CR. Commonly used drugs such as nonsteroidal anti-inflammatory drugs (NSAIDs), opioids, and corticosteroids are associated with significant side effects and are unsuitable for long-term use (22, 23). In contrast, Chinese herbal medicine, including MCHF, has demonstrated promising therapeutic effects in relieving symptoms, modulating inflammation, and promoting nerve repair in patients with CR (11, 24). However, previous studies of MCHF have often involved a combination with other adjunctive therapies, and the existing systematic reviews are not sufficiently comprehensive. Therefore, this study aimed to conduct a rigorous and comprehensive evaluation of the efficacy of MCHF by systematically analyzing placebo-controlled trials, add-on trials, and head-to-head comparisons.

We included three types of studies in this systematic review. Placebo-controlled trials aim to eliminate the placebo effect and assess the true therapeutic efficacy of MCHF. By comparing the MCHF group with the placebo group, these studies help determine whether the observed benefits are attributable to MCHF itself rather than to psychological factors or patient expectations. Add-on studies involve adding the experimental drug (MCHF) to an established standard treatment in the intervention group to evaluate the additional effect of MCHF beyond the standard therapy. Head-to-head studies refer to clinical trials that directly compare two or more active interventions without using a placebo control. In the context of CR, such studies compare MCHF with other oral medications to evaluate their relative efficacy and safety. By including these three types of studies, this review aimed to provide more robust evidence on the clinical effectiveness of MCHF and support physicians in selecting the most appropriate treatment option for patients with CR.

Pain is the primary symptom of CR and significantly impacts patients’ quality of life. Among the available tools for assessing pain intensity, the VAS is widely used due to its simplicity, reliability, validity, and broad clinical applicability (25, 26). Since most current studies report overall VAS scores, and patients often find it difficult to distinguish between neck and upper limb pain in clinical practice, the overall change in VAS after treatment will be used as the primary outcome measure in this meta-analysis. Secondary outcomes will include the JOA, NDI, SF-36, and the incidence of adverse events.

For the primary outcome measure, if high heterogeneity is detected, subgroup analyses are conducted to explore potential sources. Subgroup analyses are based on factors such as publication year, sample size, randomization method, gender ratio, and treatment duration. *p*-values for interaction (P_interaction_) are calculated to assess differences between subgroups. In addition, sensitivity analyses are performed to evaluate the robustness of the results. TSA is also applied to the primary outcome to determine whether the cumulative sample size of the included studies is sufficient to support a reliable and conclusive result. TSA helps minimize the risk of Type I and Type II errors by adjusting for random error and heterogeneity in cumulative meta-analyses. However, it is important to note that TSA has certain limitations. The required information size (RIS) may not always reflect the true effect of the intervention. Furthermore, in cases involving small-scale trials or limited data, the RIS may not be reached, indicating the need for additional studies to confirm the reliability of the conclusions (27). A limitation of this study is that it does not include patent herbal medicines from regions outside of China, such as those from Japan or South Korea.

This study is expected to provide comprehensive evidence on the efficacy and safety of MCHF in the treatment of CR, thereby contributing to the development of evidence-based recommendations for clinical practice.

## References

[1] Akbari KK Terry THL Kanade U Choi J. Clinical outcomes following treatment of cervical Spondylotic radiculopathy with cervical posterior decompression using unilateral Biportal endoscopic technique: a single Center retrospective series of 20 patients. Int J Spine Surg. (2025) 19:19–26. doi: 10.14444/8690 39547679 PMC12053253

[2] Kong S Qian X Cai J Wang J Wang K. Percutaneous plasma disc decompression through a lower surgical approach for the treatment of cervicogenic headache in patients with cervical spondylotic radiculopathy: a retrospective cohort study. Biomed Rep. (2024) 21:152. doi: 10.3892/br.2024.1840 39247422 PMC11375622

[3] Apaydin AS Güneş M. Relationships between stenosis severity, functional limitation, pain, and quality of life in patients with cervical spondylotic radiculopathy. Turk J Med Sci. (2024) 54:727–34. doi: 10.55730/1300-0144.5842 39295627 PMC11407326

[4] Davies BM Mowforth OD Smith EK Kotter MR. Degenerative cervical myelopathy. BMJ. (2018) 360:k186. doi: 10.1136/bmj.k186 29472200 PMC6074604

[5] Luyao H Xiaoxiao Y Tianxiao F Yuandong L Wang P. Management of Cervical Spondylotic Radiculopathy: a systematic review. Global. Spine J. (2022) 12:1912–24. doi: 10.1177/21925682221075290 PMC9609507 35324370

[6] Watanabe M Chikuda H Fujiwara Y Furuya T Kanchiku T Nagoshi N . Japanese orthopaedic association (JOA) clinical practice guidelines on the Management of Cervical Spondylotic Myelopathy,2020 - secondary publication. J Orthop Sci. (2023) 28:1–45. doi: 10.1016/j.jos.2022.03.012, PMID: 35618542

[7] Zhu L Gao J Yu J Feng M Li J Wang S . Jingtong granule: a Chinese patent medicine for cervical radiculopathy. Evid Based Complement Alternat Med. (2015) 2015:158453. doi: 10.1155/2015/158453, PMID: 26064154 PMC4443761

[8] Hu J Chen F Qiu G Sun T Yang H Shen H . Jingshu Keli for treating cervical spondylotic radiculopathy: the first multicenter, randomized, controlled clinical trial. J Orthop Translat. (2020) 27:44–56. doi: 10.1016/j.jot.2020.10.010, PMID: 33376673 PMC7758457

[9] Colombo C Salvioli S Gianola S Castellini G Testa M. Traction therapy for cervical radicular syndrome is statistically significant but not clinically relevant for pain relief. A systematic literature review with meta-analysis and trial sequential analysis. J Clin Med. (2020) 9:3389. doi: 10.3390/jcm9113389 33105668 PMC7690405

[10] Wang P Zuo G Du SQ Gao TC Liu RJ Hou XZ . Meta-analysis of the therapeutic effect of acupuncture and chiropractic on cervical spondylosis radiculopathy: a systematic review and meta-analysis protocol. Medicine (Baltimore). (2020) 99:e18851. doi: 10.1097/MD.0000000000018851, PMID: 32000386 PMC7004792

[11] Sun W Zheng K Liu B Fan D Luo H Qu X . Neuroprotective potential of Gentongping in rat model of cervical Spondylotic radiculopathy targeting PPAR-γ pathway. J Immunol Res. (2017) 2017:9152960. doi: 10.1155/2017/9152960, PMID: 29230425 PMC5694586

[12] Shamseer L Moher D Clarke M Ghersi D Liberati A Petticrew M . Preferred reporting items for systematic review and meta-analysis protocols (PRISMA-P) 2015: elaboration and explanation. BMJ. (2015) 349:g7647. doi: 10.1136/bmj.g7647, PMID: 25555855

[13] Bono CM Ghiselli G Gilbert TJ Kreiner DS Reitman C Summers JT . An evidence-based clinical guideline for the diagnosis and treatment of cervical radiculopathy from degenerative disorders. Spine J. (2011) 11:64–72. doi: 10.1016/j.spinee.2010.10.023, PMID: 21168100

[14] Higgins JPT Savović J Page MJ Elbers RG. Sterne JAC. Chapter 8: assessing risk of bias in a randomized trial. In: Higgins JPT, Thomas J, Chandler J, Cumpston M, Li T, Page MJ, Welch VA (editors). Cochrane Handbook for Systematic Reviews of Interventions version. (2019) 6:5.

[15] Sterne JAC Savović J Page MJ Elbers RG Blencowe NS Boutron I . RoB 2: a revised tool for assessing risk of bias in randomised trials. BMJ. (2019) 366:l4898. doi: 10.1136/bmj.l4898, PMID: 31462531

[16] McGuinness LA Higgins JPT. Risk-of-bias VISualization (robvis): an R package and shiny web app for visualizing risk-of-bias assessments. Res Synth Methods. (2021) 12:55–61. doi: 10.1002/jrsm.1411, PMID: 32336025

[17] GRADE Working Group. GRADE handbook for grading quality of evidence and strength of recommendations. (2013) [cited 01 Apr 2025]. Available online at: https://gdt.gradepro.org/app/handbook/handbook.html.

[18] Guyatt G Oxman AD Akl EA Kunz R Vist G Brozek J . GRADE guidelines: 1. Introduction-GRADE evidence profiles and summary of findings tables. J Clin Epidemiol. (2011) 64:383–94. doi: 10.1016/j.jclinepi.2010.04.026, PMID: 21195583

[19] Thorlund K Engstrøm J Wetterslev J Brok J Imberger G Gluud C (2017). User manual for trial sequential analysis (TSA) [pdf]. 2nd ed. Copenhagen: Copenhagen Trial Unit, pp. 1–119.

[20] Childress MA Becker BA. Nonoperative Management of Cervical Radiculopathy. Am Fam Physician. (2016) 93:746–54. PMID: 27175952

[21] Salt E Wright C Kelly S Dean A. A systematic literature review on the effectiveness of non-invasive therapy for cervicobrachial pain. Man Ther. (2011) 16:53–65. doi: 10.1016/j.math.2010.09.005, PMID: 21075037

[22] Onks CA Billy G. Evaluation and treatment of cervical radiculopathy. Prim Care. (2013) 40:837–48. doi: 10.1016/j.pop.2013.08.004, PMID: 24209721

[23] Baselyous Y De Cocinis M Ibrahim M Kalra A Yacoub R Ahmed R. Potentially inappropriate concomitant medicine use with the selective COX-2 inhibitor celecoxib: analysis and comparison of spontaneous adverse event reports from Australia, Canada and the USA. Expert Opin Drug Saf. (2019) 18:153–61. doi: 10.1080/14740338.2019.1589447, PMID: 30929580

[24] Li W Yao C Zhou Y Chen S. Changes of Endothelin-1 and calcitonin gene-related peptide concentrations in patients with cervical radiculopathy after wrist-ankle acupuncture-Moxibustion and hot compression with Chinese herbal medicine. Genet Res. (2021) 2021:1–7. doi: 10.1155/2021/5433742, PMID: 35002538 PMC8710150

[25] Haefeli M Elfering A. Pain assessment. Eur Spine J. (2006) 15:S17–24. doi: 10.1007/s00586-005-1044-x 16320034 PMC3454549

[26] Åström M Thet Lwin ZM Teni FS Burström K Berg J. Use of the visual analogue scale for health state valuation: a scoping review. Qual Life Res. (2023) 32:2719–29. doi: 10.1007/s11136-023-03411-3, PMID: 37029258 PMC10474194

[27] Kang H. Trial sequential analysis: novel approach for meta-analysis. Anesth Pain Med. (2021) 16:138–50. doi: 10.17085/apm.21038, PMID: 33940767 PMC8107247

